# Supplementary material for: Graded 2D/3D Perovskite Hetero-Structured Films with Suppressed Interfacial Recombination for Efficient and Stable Solar Cells via DABr Treatment
Source: Molecules. 2023 Feb 7;28(4):1592. doi: 10.3390/molecules28041592 (PMC9964978; doi:10.3390/molecules28041592)
Supplement: Supplementary file 1 [file molecules-28-01592-s001.zip › molecules-2185835-supplementary.pdf]

Supporting Information data

Graded 2D/3D Perovskite Hetero-Structured Films with Suppressed Interfacial Recombination for Efficient and Stable Solar Cells via DABr Treatment

Muhammad Mateen [1], Hongxi Shi [1], Hao Huang [1], Ziyu Li [1], Waseem Ahmad [2], Muhammad Rafiq [3], Usman Ali Shah [4], Sajid Sajid [5], Yingke Ren [6], Jongee Park [7], Dan Chi [1,*], Zhangbo Lu [1] and Shihua Huang [1,*]

[1] Provincial Key Laboratory of Solid-State Optoelectronic Devices, Zhejiang Normal University, Jinhua 321004, China; bozdarmateen@yahoo.com (M.M.); shongxi@zjnu.edu.cn (H.S.); huanghao@zjnu.edu.cn (H.H.); liziyu@zjnu.edu.cn (Z.L.); luzhangbo@zjnu.edu.cn (Z.L.)

[2] Division of Science and Technology, Department of Physics, University of Education Campus Dera Ghazi Khan, Multan 32200, Pakistan; boslib@yahoo.com

[3] Institute of Biomedical Materials and Engineering, College of Materials Science and Engineering, Qingdao University, Qingdao 266071, China; rafiqqaisrani92@gmail.com

[4] Department of Physics and Astronomy, University of Florence, via Giovanni Sansone1, Sesto Fiorentino, I-50019, Italy; usman.pir59@gmail.com

[5] Department of Chemical & Petroleum Engineering, United Arab Emirates University, Al Ain P.O. Box 15551, United Arab Emirates; fary_sajjo@yahoo.com

[6] Hebei Provincial Key Laboratory of Photoelectric Control on Surface and Interface, College of Science, Hebei University of Science and Technology, Shijiazhuang 050018, China; renyingke@yeah.net

[7] Department of Metallurgical and Materials Engineering, Atilim University, Ankara 06836, Turkey; jongee.park@atilim.edu.tr

* Correspondence: chidan@zjnu.edu.cn (D.C.); huangshihua@zjnu.cn (S.H.)

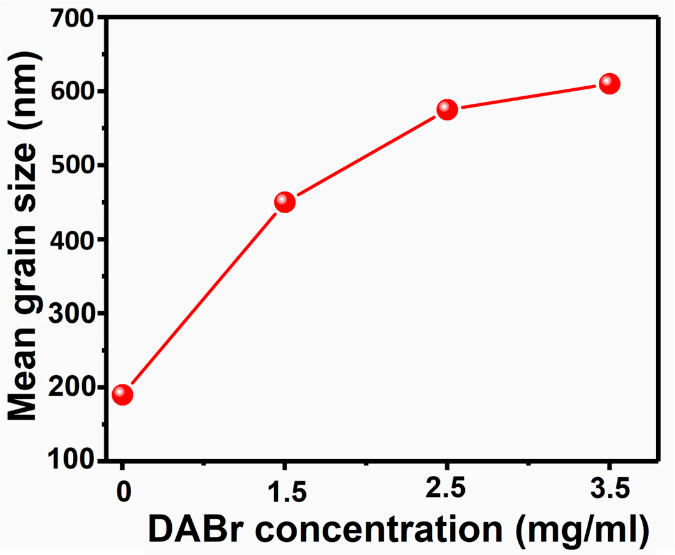

Figure S1. Grain size distribution of control, DABr-1.5, DABr-2.5, and DABr-3.5 treated 2D-3D perovskites.

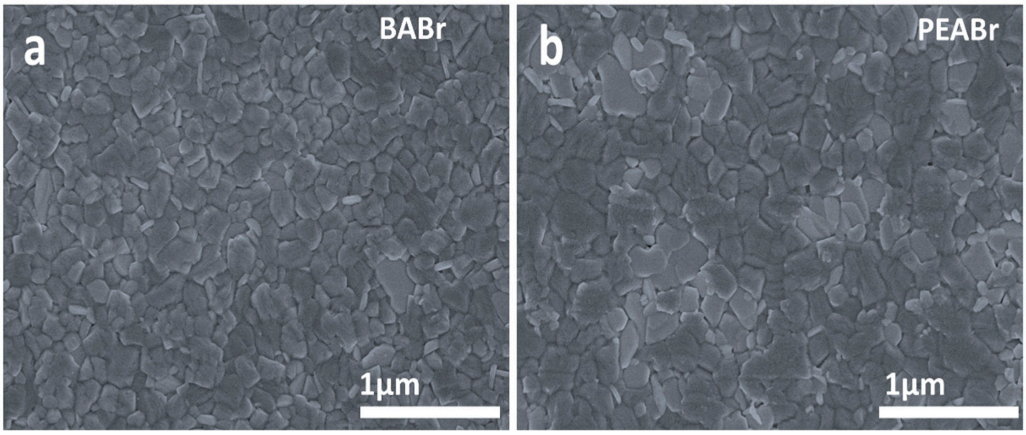

Figure S2. Surface SEM images of (a) PEABr-2.5 and (b) BABr-2.5 post-treated 2D-3D stacked perovskite films.

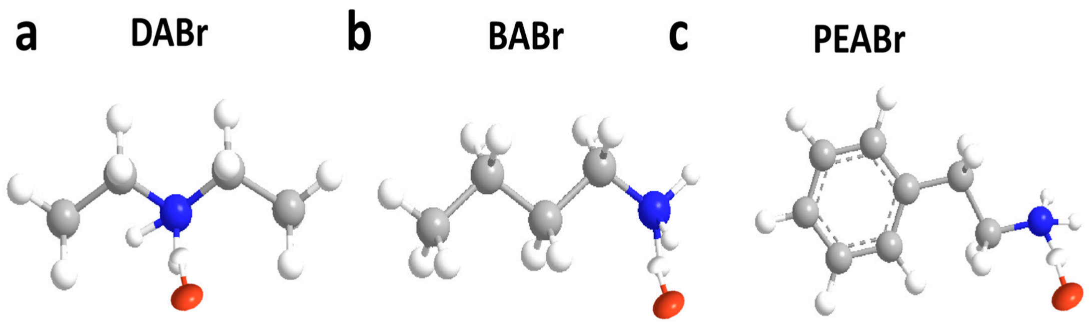

Figure S3. The schematic illustrations of the molecular structures for DABr, BABr, and PEABr.

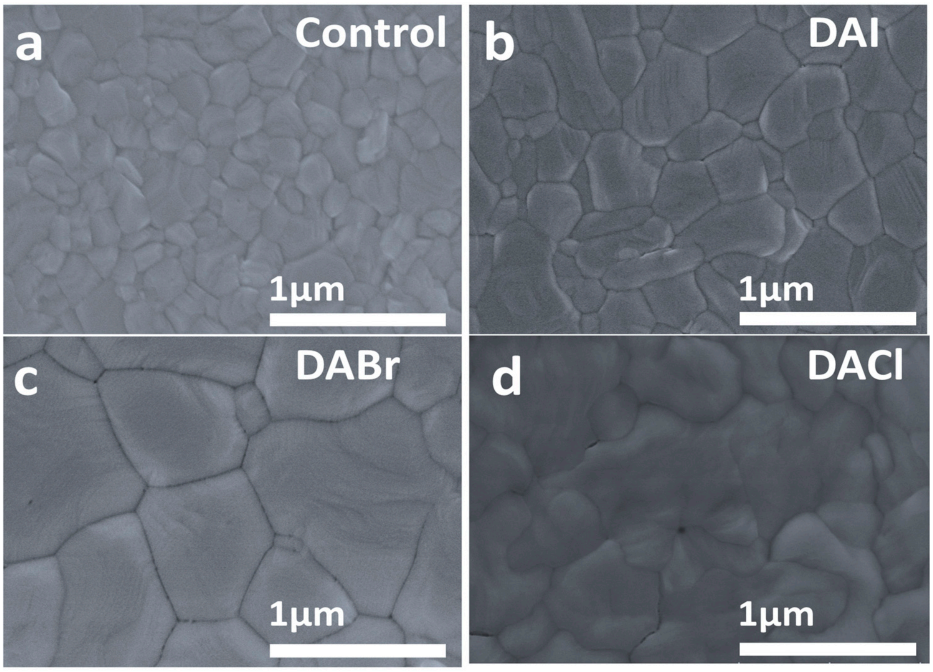

Figure S4. Top view of SEM images for (a) control (b) DAI-2.5, (c) DABr-2.5, and (d) DACl-2.5 post-treated 2D-3D perovskite films.

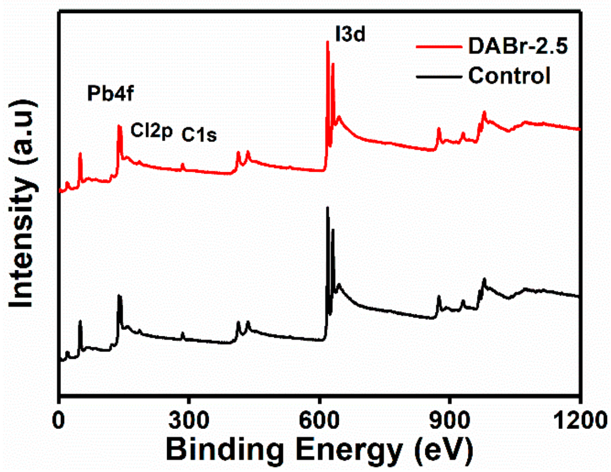

Figure S5. XPS core spectra of pure $MAPbI_3$ perovskite film and perovskite film post-treated with DABr-2.5.

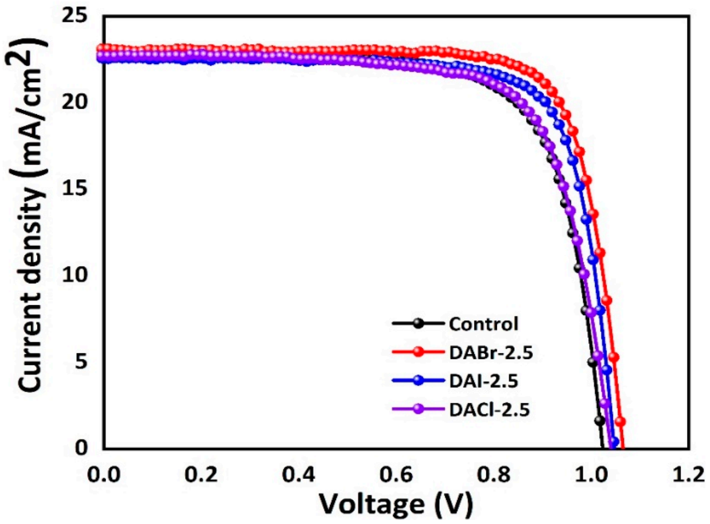

Figure S6. *J-V* characteristic curves of the perovskite devices consist of control, DABr-2.5, DAI-2.5, and DACl-2.5 treated films. The measurements were conducted during reverse scanning under one sun illumination at 100 mW·cm^{-2}.

Table S1. Fitted parameters of the TRPL curves of pristine 3D-$MAPbI_3$ perovskite and DABr-2.5 treated 2D-3D perovskite films.

Samples	τ_1 (ns)	A_1	τ_2 (ns)	A_2	T average (ns)
Control	10.09	40.67	67.24	51.00	63.34
DABr-2.5	34.59	43.40	169.35	68.30	145.36

Table S2. Performance parameters obtained from the *J-V* characteristics of the perovskite devices consist of control, DABr-2.5, DAI-2.5, and DACl-2.5 treated films.

Perovskite	Jsc ($mA.cm^{-2}$)	Voc (V)	FF (%)	PEC (%)
Control	22.78	1.02	72.75	16.98
DABr-2.5	23.15	1.06	77.85	19.10
DAI-2.5	22.94	1.05	76.30	18.02
DACl-2.5	22.46	1.03	73.60	17.20
